# Inguinal Squamous Cell Carcinoma Presenting as a Marjolin Ulcer in the Absence of Mesh Infection: A Case Report

**DOI:** 10.7759/cureus.39002

**Published:** 2023-05-14

**Authors:** Kaveh Mozafari, Shaniah S Holder, Dil Siwakoti, Alex C Lieberman, Frederick Tiesenga

**Affiliations:** 1 Surgery, West Suburban Medical Center, Oak Park, USA; 2 Medicine, St. George's University School of Medicine, Saint George's, GRD; 3 Medicine, American University of Barbados School of Medicine, Bridgetown, BRB; 4 Surgery, St. George's University School of Medicine, Saint George's, GRD; 5 Surgery, St. George's University School of Medicine, Saint George's, USA; 6 General Surgery, West Suburban Medical Center, Oak Park, USA

**Keywords:** mesh infection, marjolin ulcer, inguinal mesh, inguinal hernia, squamous cell carcinoma, bilateral inguinal hernia repair, general surgery, deep mesh infection, inguinal hernia repair, squamous cells carcinoma

## Abstract

Inguinal hernias are the most common type of hernias, and most are repaired surgically with mesh placement to prevent future reoccurrence. Mesh infection and hernia recurrence are some of the rare complications of mesh placement, and chronic mesh infections increase the risk of squamous cell carcinoma at the site. Squamous cell carcinoma (SCC) in the setting of a mesh infection presents in a similar manner to a Marjolin ulcer and is treated with the removal of the tumor and degraded infected mesh. However, in this case, the patient presented atypically with an absence of mesh involvement. This report aims to explore the etiology of SCC due to mesh infections as well as describe the perplexing case of inguinal SCC in the absence of mesh involvement.

## Introduction

Inguinal hernias affect 25% of men as opposed to less than 2% of women. The most common type is an indirect hernia, which often occurs on the right side, possibly attributed to more unhurried closure of patent processus vaginalis on the right side when compared to the left side. One possible predisposing factor for the development of an inguinal hernia in adulthood is patent processus vaginalis [1]. The two categories of inguinal hernias, direct and indirect, are distinguished by the path the abdominal contents protrude through. In an indirect hernia, the protrusion passes through the internal inguinal ring and into the inguinal canal lateral to the inferior epigastric vessels. As in the case of a direct hernia, the abdominal content obtrudes via transversalis fascia within Hesselbach’s triangle. Typical presentation includes a bulging mass that may or may not be painful and is normally repaired surgically when symptomatic due to the increased risk of bowel obstruction, strangulation, and ischemia; however, elective surgery is also common to prevent noted emergency situations [2]. The use of mesh in hernia repair has become popular over the decades due to its decreased hernia recurrence rate (in some studies, from 66/100,000 person-years to 26/100,000 person-years but the data is not consistent everywhere in the world); however, it has been associated with many infectious and non-infectious complications. Complications occur in 8-10% of patients and include seromas, hematomas, surgical site infections, mesh migration and erosion, and mesh infection [3]. Although studies infer that there is no increased risk of subsequent cancer development in patients with mesh-based hernia repair, there is an association between mesh infections and squamous cell carcinoma (SCC) development [4]. Mesh infections are rare; therefore, there is a paucity of literature exploring the association of SCC in the setting of mesh placement. 

SCC is one of the most common dermatologic cancers and affects approximately 3.3 million Americans [5]. Risk factors include excess ultraviolet (UV) radiation, immunosuppression, and some genetic disorders, and surgical excision and chemotherapy continue to be the mainstay modes of treatment [6]. SCC may arise anywhere on the body; however, it typically presents in sun-exposed areas such as the face, scalp, and dorsal aspect of the forearms and hands [7]. Features of SCC may vary from a fleshy to erythematous appearance and is often associated with hyperkeratosis, which manifests as scaly, crusty tissue coupled with ulceration due to cellular invasion and degradation [7]. Lesions are commonly plaque-like but can be flat or nodular and occasionally, patients may complain of pain due to perineural invasion [7]. 

Most cases of SCC in the setting of mesh placement arise due to long-term mesh infections. A report by Birolini et al. found that SCC, due to chronic mesh infection, presents like a Marjolin ulcer and tends to spread rapidly [8]. The mainstay treatment of SCC due to mesh infection is radical surgical excision of the tumor with the removal of the infected mesh and adjuvant chemotherapy if required [8]. However, this is the first reported case of inguinal SCC that presented as a Marjolin ulcer in a patient with a history of mesh placement but in the absence of a mesh infection.

## Case presentation

The patient, in this case, is an 88-year-old male who presented to the emergency department (ED) with complaints of pain and swelling in his right inguinal region for the past few hours. The patient has a past surgical history significant for bilateral inguinal hernia repair with mesh placement three years prior. He denied nausea, vomiting, fever, chills, or night sweats but reported discharge from the site. On physical examination, there was an erythematous right groin scar of inguinal incision with staggered dehiscence and an ulcerative mass with foul-smelling mucopurulent drainage that was tender to palpation without any inguinal region lymphadenopathy. 

**Figure 1:**
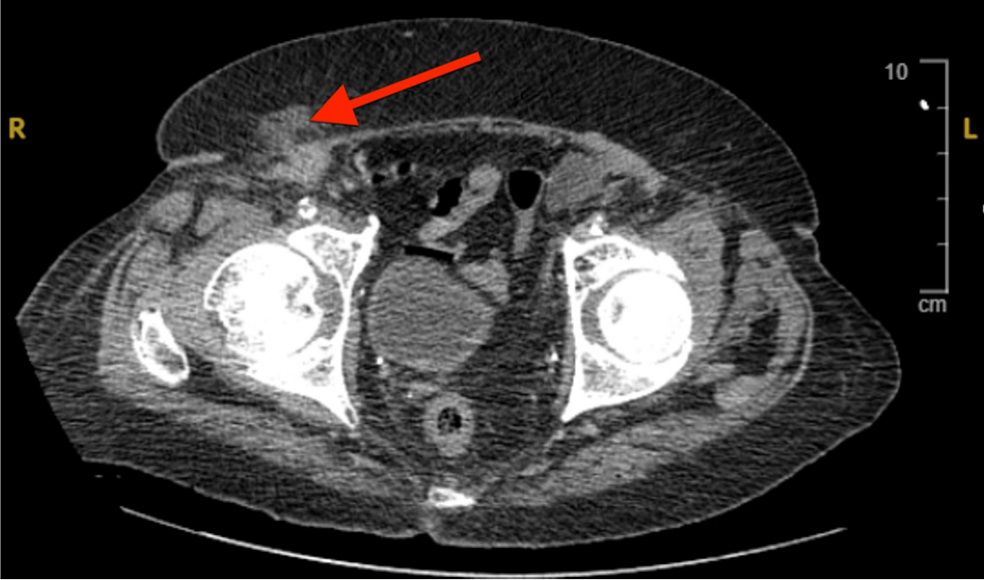
Hyperdense tissue visualized in the right inguinal canal

Laboratory values were significant for leukocytosis with neutrophil predominance with values of 12,700 WBC/μL and 9200 cells/μL, respectively. These findings were suspicious for hernia recurrence and possible mesh infection. The patient was placed on prophylactic amoxicillin-clavulanate and imaging with computed tomography (CT) scan was conducted (Figure 1). Findings were notable for the presence of an irregular enhancement in the right inguinal canal.

Due to the positive surgical history of inguinal mesh placement, clinical features, and ulcerative mass with purulent drainage, the patient was suspected of having an inguinal hernia mesh infection and was scheduled for surgical groin exploration two days after admission to the hospital based on clinical and radiographic evidence.

The patient was under general anesthesia. Upon incision of the surrounding tissue, two apertures were located in the inguinal crease, both separated by a layer of skin. Incisions were made over the two draining sinus tracts, and a significant amount of beefy hypergranulation tissue was visualized and excised. Exploration was extended all the way down to the fascia and further towards the direction of the pubis symphysis. It was found that the bone was covered with muscle, and there was no evidence of pus or mesh protruding from under the muscle. After further exploration, it was concluded that the infection was simply on the subcutaneous level, and the necrotic, hypergranulated tissue was excised and sent to pathology. The patient tolerated the procedure well and had no postoperative complications. 

Pathology results were remarkable for invasive moderately differentiated squamous cell carcinoma. The oncology department was consulted, and CT of the abdomen and pelvis was conducted; results were negative for a primary site of malignancy or metastasis. 

The patient was informed of his diagnosis and followed up with wound care and oncology for treatment.

## Discussion

Surgical repair is the definitive therapeutic management for all symptomatic inguinal hernias. Some methods, such as the Bassani, Shouldice, and McVay repairs use the native tissue to close the defect; however, there is a lower recurrence rate reported with prosthetic tissue use [9]. Prosthetic repairs consist of the use of mesh in the preperitoneal space to block the defect [9]. There is an increased risk of hernia repair complications in persons with advanced age, obesity, tobacco use, and the presence of bilateral inguinal hernias [10]. 

Mesh infections are an extremely rare complication and account for 1-8% [11]. The etiology of mesh infections is not well known; however, studies suggest that exogenous contamination leads to bacterial invasion and growth, resulting in an infection within the first few weeks postoperatively [12]. Mesh-induced intestinal erosion and fistula formation may cause late-onset mesh graft infections due to the hematogenous or direct spread of intestinal flora into the mesh [12]. Patients with mesh infections typically present with fever, malaise, and chills. Physical examination is normally significant for tenderness, erythema, and occasionally a draining fistula or mass if herniation has reoccurred [3]. Mesh infections are associated with chronic inflammation secondary to infection, which can precipitate neoplastic changes in tissue leading to SCC [8]. 

SCC in the setting of chronic mesh infection presents like a Margolin ulcer with a foul-smelling ulcerative or indurated lesion with rolled elevated margins [10,13]. Other features include granulation tissue, bleeding, and regional lymphadenopathy [13]. The ulcer may also become superinfected, leading to fever, chills, and leukocytosis, which may be the initial presenting symptom [13]. The patient, in this case, had an ulcerative lesion with mucopurulent discharge and a large amount of granulation tissue. His laboratory tests were also significant for leukocytosis with neutrophil predominance, which is common in superinfected Marjolin ulcers. Marjolin ulcer is a cutaneous malignancy that normally appears in chronic wounds, burns, or scars [13]. They arise due to chronic irritation, local damage, and repeated re-epithelization and can deteriorate into poorly differentiated SCC on histopathology [9]. In this case, the patient’s biopsy was significant for moderately differentiated SCC on histopathology. 

Imaging with CT is useful in identifying fluid collections or edema with fat stranding around the mesh, indicating an underlying mesh infection [12]. The features of a Marjolin ulcer on CT include bone destruction, soft tissue mass, and periosteal reaction [14]. Soft tissue masses appear as irregular and nodular enhancing, which was visualized on CT in this patient [14]. SCC in the setting of mesh infection is typically treated with radical surgical excision of the infected mesh and tissue with the use of chemotherapy in the case of metastasis [8]. 

During the groin exploration operation, it was noted that the infection was superficial and did not involve the muscle, bone, or mesh, which was atypical for the histopathological findings of SCC in the setting of mesh placement. There are no known cases of Marjolin ulcer or SCC in the setting of inguinal mesh placement in the absence of mesh infection. Chronic irritation of the epithelium could have precipitated this neoplastic transformation, and whether this was due to the underlying mesh, the presence of scar tissue, or another cause is not known. Although mesh placement is not usually associated with secondary carcinoma development, this may be a rare occurrence that requires further research.

## Conclusions

Inguinal SCC is a rare complication of mesh placement and typically occurs in the setting of mesh infections due to secondary inflammation. There are no previously documented cases of developing SCC after mesh placement in the absence of a mesh infection. Our case presents an unexplored complication that requires further research. Most cases of SCC in the setting of mesh infection present like a Marjolin ulcer with an ulcerative lesion with granulation tissue that may become superinfected, leading to leukocytosis. 

The patient in this report presented with all the clinical features of a Marjolin ulcer; however, there were no signs of mesh infection. Physicians should be aware of the increased risk of SCC in patients with long-term mesh infection and also in patients who present with features of Marjolin ulcer with a history of mesh placement. Early and appropriate diagnostic and therapeutic management is paramount for improving the patient’s quality of life.
